# Neurobiological mechanisms of mood disorders: Stress vulnerability and resilience

**DOI:** 10.3389/fnbeh.2022.1006836

**Published:** 2022-10-28

**Authors:** Clairton Marcolongo-Pereira, Fernanda Cristina de Abreu Quintela Castro, Rafael Mazioli Barcelos, Kelly Cristina Mota Braga Chiepe, Joamyr Victor Rossoni Junior, Roberta Passamani Ambrosio, Orlando Chiarelli-Neto, Ana Paula Pesarico

**Affiliations:** ^1^Coordenadoria de Pesquisa, Pós-Graduação e Extensão (CEPEG), Centro Universitário do Espírito Santo (UNESC), Colatina, Brazil; ^2^Curso de Medicina, Universidade Federal do Pampa (Unipampa), Bagé, Brazil

**Keywords:** stress, depression, anxiety, resilience, vulnerability

## Abstract

Stress is an important factor in the development of several human pathologies. The response of rodents and humans to stress depends on many factors; some people and rodents develop stress-related mood disorders, such as depression and anxiety in humans, depression-like and anxiety-like behavior in mice and rats, while others report no new psychological symptoms in response to chronic or acute stress, and are considered susceptible and resilient to stress, respectively. Resilience is defined as the ability to thrive in the face of adversity and is a learned process that can help protect against occupational stressors and mental illnesses. There is growing interest in the underlying mechanisms involved in resilience and vulnerability to depression caused by stress, and some studies have demonstrated that individual variability in the way animals and humans respond to stress depends on several mechanisms, such as oxidative stress, neuronal plasticity, immunology and genetic factors, among others not discussed in this review, this review provides a general overview about this mechanism.

## Introduction

Depression is a prevalent, chronic, and recurrent mental disorder that affects more than 280 million people and is one of the leading causes of disability worldwide (Evans-Lacko et al., 2018). This disorder has significant social and economic consequences; the annual cost of depression is estimated to be approximately 326 billion dollars in the United States (Greenberg et al., 2021). Although patients present several characteristics, declined interest and alterations in appetite, sleep, and energy levels are more specifically developed by depressive people (Malhi and Mann, 2018). Furthermore, depression can exist as a comorbidity for disorders such as obesity and Alzheimer’s disease (Ryu et al., 2017; Wang et al., 2022), migraine, epilepsy, and sleep disorders (Steffen et al., 2020).

The development of depression is influenced by many factors including psychological, environmental, and biological factors. Stress is an environmental factor and one of the most important factors responsible for depression (Russo et al., 2012), while work problems and family disagreements are two domains considered to be involved (Chintoh and Trevor Young, 2016; McCarty, 2020). However, there is a fundamental question: Why are some people more resilient than others?

Many rodent models have been used to investigate the effects of stress on biochemical and molecular pathways in a multitude of human pathologies. Some experimental stress models have been used to investigate the reason for resilience to stress, as every mouse experiencing stress does not develop features of depression-like or anxiety-like behavior (Yao et al., 2016; Pesarico et al., 2017; Yang et al., 2017), and it is important to note that the same situation occurs with humans (Alim et al., 2008; Schetter and Dolbier, 2011; Pietrzak and Cook, 2013).

The word “resilience” denotes the ability to withstand or recover quickly from difficult conditions (Fletcher and Sarkar, 2013) or may be considered an outcome after experiencing adversity (Masten, 2001). The investigation of resilience as resistance to stress began in the 1970s in a study of children capable of normal development, despite the disadvantages and adversities (Masten, 2001). It is important to investigate the factors that are correlated with resilience because 70% of patients with depression are not treated with first-line antidepressant drugs. Furthermore, the mechanisms of resilience provide a possible alternative to traditional antidepressants.

The neural and molecular pathways involved in depression and resilience to stress have been investigated in laboratory animals and humans. Oxidative stress is defined as an imbalance between the production of reactive oxygen and nitrogen species (ROS/RNS) and the antioxidant capacity of the cell. These substances are necessary for normal brain function as well as in the pathogenesis of mood disorders such as depression, because exposure to oxidative stress in the brain causes damage to neuronal deoxyribonucleic acid (DNA), among other molecules (Salim, 2017; Majnarić et al., 2021). Furthermore, a study demonstrated that substances involved in cellular defense against oxidation can confer stress resilience in adult mice (Yao et al., 2016).

**FIGURE 1:**
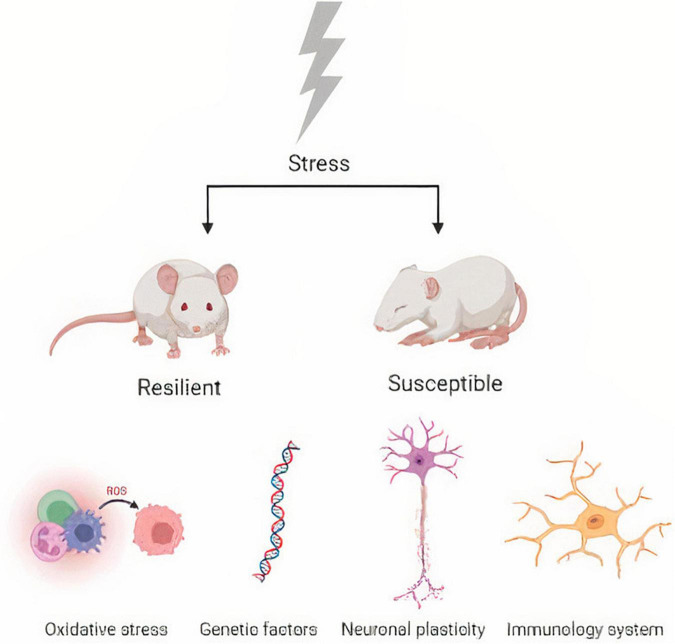
Biological mechanisms of stress susceptibility and resilience.

Another important pathway in depression and resilience to stress is the inflammatory response, and a study has demonstrated that patients exposed to stress and depression exhibit increased inflammatory markers (Danese et al., 2008). Further, another study showed that IL-1β genes could be regulated and are good markers for identifying susceptibility and resilience to stress (Wood et al., 2015). Synaptic plasticity (Wang et al., 2018), immunity (Dantzer et al., 2018), neuroendocrine mechanisms (Ménard et al., 2017) and gut microbiota (Yang et al., 2017) are other pathways involved in depression that promote stress resilience. For example, important data demonstrate that germ-free mice have been shown to be more susceptible to depression (Zheng et al., 2016) (Figure 1).

The present review explores biological and social aspects related to depression and resilience to stress and discusses the importance of oxidative stress and other mechanisms involved in resilience to stress and mood disorders. For readers unfamiliar with this area, this review provides a general overview of the various neural pathways that contribute to depression and resilience to stress.

## Relation of biological and social aspects with individuality of response to stress

Dysregulation of the stress response can lead to psychiatric illnesses; however, not all individuals exposed to stress develop some type of psychiatric disorder. Stress results from environmental adversity, and some individuals develop stress-resilience mechanisms without developing persistent psychopathology. Consequently, the investigation of the stress response has increased significantly in recent decades.

The social environment can be a source of stressors and be beneficial in reducing the biological effects of stress. Many mental and physical illnesses are triggered by exposure to stressors, resulting in a negative association with stress. However, the activation of this response is important for the development of adaptive capacity and survival of individuals (Wood and Bhatnagar, 2015). The impact of a stressor agent is determined by the characteristics of this agent, as well as the abilities of the organism in relation to this agent (Korte et al., 2005; Osório et al., 2017).

These abilities of the organism (body skills) involve physiological and behavioral reactions that determine the coping style, which, in turn, depends on genetic and epigenetic bases, mainly those of social origin (Veenema et al., 2003; Wood and Bhatnagar, 2015). These bases exert a great influence on basal levels of cortisol as well as on the individual’s reaction to stress situations (Veenema et al., 2003; Osório et al., 2017).

One study revealed a positive association between good emotions and resilience, indicating that coping strategies partially mediate this relationship (Gloria and Steinhardt, 2016). Another prospective longitudinal study provided evidence that individual differences in stress resilience are predicted by both lower levels of negative emotions and higher levels of responsiveness in a positive direction prior to exposure to stressors, suggesting that coping strategies are essential to minimize stress and its impact (Galatzer-Levy et al., 2013).

There is an important distinction to be made between the potentially harmful and protective effects of stress mediators, leading to the introduction of two terms: First, “allostasis,” which refers to maintaining homeostasis by actively releasing stress hormones and regulation of changes in the set point and efficient prevention of errors by prediction and feedforward mechanisms, in combination with switching the coping strategy depending on the environment (McEwen, 2007; Schulkin and Sterling, 2019; Guidi et al., 2021); In addition, “allostatic load or overload” refers to the cumulative burden of chronic stress and life events to the wear and tear on the body and brain caused by allostasis, especially when mediators are not turned off when stress ends and not turned on properly when needed. It involves the interaction of different physiological systems with varying degrees of activity (McEwen, 2007; Guidi et al., 2021).

Allostasis involves autonomic, inflammatory, metabolic, and neuromodulatory mediators, which interact with each other and promote adaptive capacity. When this adaptive mechanism is regulated efficiently and infrequently and can be turned “on and off,” the organism can efficiently deal with challenges (McEwen and Wingfield, 2010; McEwen et al., 2015b). However, if the allostatic system is overstimulated or does not respond appropriately, a system imbalance can occur, favoring the emergence of diseases. Therefore, coping strategies determine whether the response will result in positive stress, with a satisfactory outcome, or there would be negative consequences (Veenema et al., 2003; Wood and Bhatnagar, 2015).

Individuals who lack the ability to deal with stressful situations are more vulnerable, resulting in inappropriate activation of allostatic systems and unbalancing the functioning of other physiological systems (McEwen and Wingfield, 2010; Bogeska, 2021). Thus, stress is often associated with various physical pathologies and mental disorders (Wood and Bhatnagar, 2015; Osório et al., 2017).

The social environment can be a source of stressors, or be beneficial to individuals during crisis situations, reducing the harmful effects of stress (Creel, 2001; Beery and Kaufer, 2015; Bogeska, 2021).

At the cognitive level, this support promotes the reduction of perceived stress, and at a physiological level, it reduces the functioning of the hypothalamic-pituitary-adrenal (HPA) axis, sympathetic autonomic nervous system, and inflammatory response (Seeman and McEwen, 1996). These effects can occur together or at different times, and vary depending on individual genetic history, experience, sex, species, and other factors (Beery and Kaufer, 2015).

Stressful situations alter social interactions while moderating the effects of stressors. Stress resilience may vary depending on the social environment early in life or it may arise from the mitigating effects of positive or even negative social interactions (Beery and Kaufer, 2015). Some studies have demonstrated that individual factors and the quality of social support relationships promote an individual’s wellbeing, directly influencing the reduction of stress response and preventing the development of pathologies (Seeman and McEwen, 1996; Wood and Bhatnagar, 2015).

## Depression and resilience to stress: Genetic factors

Depression and resilience involves a complex interplay of biological, social and psychological factors, so there are many pathways that lead to resilient functioning and advances in molecular biology and genetic knowledge have allowed the advancement of resilience studies in recent years to determine the interaction of genes with experience (Hodes and Gau, 2016). There is substantial evidence that personality traits such as intelligence and personality are inherited (Bouchard, 2004). According to the first law of genetics, all human characteristics, including psychological characteristics, are heritable (Turkheimer, 2000; Johnson et al., 2010). A combination of genetics and environment determines an individual’s vulnerability to stress. Trauma, abandonment, and other early adversities can adversely affect stress-coping mechanisms, which can become abnormal (Nold et al., 2021).

In the organic response to stress, several components work together, such as psychological, immunological, neurological, and energetic, causing changes in vital parameters and, in a chronic manner, systemic diseases (Nold et al., 2021).

Among the genetic factors that influence susceptibility or resistance, serotonin-transporter-linked promoter region (5-HTTLPR) appears to play a central role in the stress regulation process (Sen et al., 2004; Takano et al., 2007; Wankerl et al., 2010). Several studies have linked 5-HTTLPR, a degenerate repeat polymorphic region of the gene encoding the serotonin transporter (5-HTT), directly to stress and mental illness (Lesch et al., 1996). When analyzing the patterns of stress reactivity, it was observed that individuals homozygous for the short allele had higher levels of endogenous cortisol during and after exposure to stressors. However, individuals with at least one long allele demonstrated a slight decrease in hormone levels over the course of the study period, suggesting that the organic response to stress is caused by an association between the serotonin transporter gene and external factors (Gotlib et al., 2008).

Studies have shown genetic alterations in the proteins for example, the activation of sirtuin 1, a protein that regulates many biological processes, including inflammation and is associated with depression. In the hippocampus of mice, it improves depressive-like behavior induced by chronic unpredictable mild stress (Duan et al., 2020). Moreover, similar stress increases Tacr2 expression, a gene responsible for the modulation of emotional processes, in a different region, the hypothalamus (Xiang et al., 2019) and elevates the expression of the proinflammatory cytokine IL-17 in the prefrontal cortex of mice (Kunisawa et al., 2022).

Furthermore, researchers have shown that people with childhood trauma express FKBP5, the gene responsible for encoding a binding protein of the same name, after traumatic events (Nold et al., 2021). A single nucleotide polymorphism in this gene can result in two alleles: the A/T allele is associated with greater negative effects of stress, and the G/C allele is associated with stress resilience (Zannas and Binder, 2014). A study with rats that experienced adversity in the first stages of life in an induced way showed the presence of both A/T and G/C alleles, and demonstrated the effect of the polymorphisms on their behavior. In addition to exhibiting decreased activity and reduced ability to adapt to their environment, mice expressing the A/T allele showed increased expression of genes related to cellular respiration, indicating a need for more energy (Nold et al., 2022).

The monoamine oxidase A (MAOA) gene was associated with a moderating effect of child maltreatment on the development of antisocial behavior in adult male subjects. The effect on antisocial behavior in adulthood was lower among males with high MAOA than among those with low MAOA activity (Caspi et al., 2002).

In addition, genes related to the HPA axis may influence the risk of depressive and posttraumatic stress symptoms contingent on the experience of child abuse (Gillespie et al., 2009). The social defeat stress, an important animal model, which mice and rats are exposed to repeated social stress causes biological alterations, such as changes in corticosterone levels and it is can be related with genetic alterations (Ajayi et al., 2022; Avgustinovich et al., 2022).

Furthermore, other preclinical studies indicate that modulation of the HPA axis function during stress response promotes a resilient phenotype (Plotsky and Meaney, 1993; Weaver et al., 2005). Activation of the HPA axis affects brain functioning to ensure a proper behavioral response to the stressor; however, stress-induced (mal) adaptation of the HPA axis functional maturation may provide a mechanistic basis for the altered stress susceptibility later in life (van Bodegom et al., 2017).

Therefore, stressors alter gene expression through multiple mechanisms, including the direct effects of glucocorticoid gene transcription, such as activation of epigenetic mechanisms, in which histone modifications and methylation of CpG residues in DNA play a role in the repression and activation of genetic factors, including retrotransposons (Meaney and Szyf, 2005; Hunter et al., 2015).

These findings suggest that genetic factors influence vulnerability and resilience to stress. Gene and environment interactions affect critical periods of emotional neural system development, differentially mediating vulnerability and resilience (Gillespie et al., 2009). Nevertheless, more research is needed to uncover the complex interaction between genetics and abnormal stressors that can lead to disease vulnerability, including psychiatric disorders.

## Depression and resilience to stress: Synaptic plasticity

Many mediators and intracellular processes are involved in brain changes during stress and recovery from stressful experiences (McEwen, 2007, 2010). Taking into account multiple mediators, including the cell surface, cytoskeleton, epigenetic regulation, and non-genomic mechanisms, the brain perceives and adapts to social and physical stressors (McEwen et al., 2015a; McEwen, 2017). When stress results in structural remodeling of the neural architecture, it may be a successful sign of adaptation, or it may indicate a failure of the resilience process (McEwen, 2008; McEwen et al., 2015a).

Synaptic plasticity is a pathway in which neural activity generated by a sensory and motor experience can affect brain function by changing synaptic transmission (Citri and Malenka, 2008; Kim et al., 2020) and it refers to the activity-dependent modification or efficiency of synaptic transmission, which has been proposed to play a central role in the brain’s ability to incorporate transient experiences (Citri and Malenka, 2008; Kim et al., 2020). Furthermore, evidence suggests that modifications in synaptic plasticity mechanisms contribute to several neuropsychiatric disorders (Fernandes and Carvalho, 2016).

Based on the understanding of the existence of plasticity, stressful events can evoke adaptive coping mechanisms that occur both in the central nervous system (CNS) and in the peripheral nervous system and are able to generate resilience (Russo et al., 2012; Pfau and Russo, 2015). Recently, a research group demonstrated that synaptic plasticity-related molecules in the hippocampus are associated with susceptibility to social defeat stress in mice (Lee et al., 2021) and another group revealed the involvement of autophagy signaling proteins; however, these proteins are increased in microglia cells of the prefrontal cortex of susceptible and resilient male mice (Sakai et al., 2022). In the hippocampus, autophagy also was reported to be associated with chronic unpredictable mild stress (Xiao et al., 2018).

In a study developed with humans, researchers demonstrated that positive emotions induce resilience to depression and anxiety and improve their overall health and it is could related with positive effects, which have been shown to induce resilience to depression through N-methyl-D-aspartate (NMDA) receptor-dependent, a protein involved in synaptic plasticity (Burgdorf et al., 2017). Corroborating with these data, animal studies have shown that chronic stress is associated with pathological glutamate excitotoxicity and synaptic dysfunction, leading to reductions in dendritic branching and spine density of pyramidal neurons, and eventually neuronal atrophy, in hippocampus and prefrontal cortex, two areas implicated in the mood disorders (Licznerski and Duman, 2013; Wang et al., 2015).

The polysialylated form of neural cell adhesion molecule (PSA-NCAM) is expressed in the CA3 and dentate gyrus (DG) regions of the hippocampus and is believed to denote the capacity for adaptive structural plasticity in several regions of the central nervous system (Seki and Arai, 1999; Theodosis et al., 1999). Repeated stress causes shrinkage of hippocampal CA3 dendrites and an increase in PSA-NCAM expression by involvement of glucocorticoid (Pham et al., 2003). Thus, while PSA-NCAM facilitates plasticity, the PSA portion also appears to limit the extent of dendritic growth (McEwen et al., 2015a).

Stress-induced changes in neural architecture are not reversible, but are forms of neuroplastic adaptations that can be impaired in mood disorders and reduced with aging (McEwen et al., 2015a). Resilience can be viewed as an active process that implies continuous adaptation without external intervention (Russo et al., 2012; Cathomas et al., 2019). Several factors influence possible responses, including the chronology of the injury, the affected site, the condition of the substrates that can assume the function, and the type of altered function. As a result, the mechanisms facilitating this plasticity differ at each moment (fast and late plasticity) depending on the function that is altered (Hernández-Muela et al., 2004; Muela et al., 2004; Chen and D’Esposito, 2014).

Brain plasticity involves two significant elements: critical periods and activity-dependent changes. The critical period is defined as the time of data reception, and significant abilities may be lost or limited if no stimulation appears at the right time (Makara-Studzińska et al., 2012). The concept of activity-dependence refers to changes that occur in the brain that can be caused by the influence of psychological, biological, or environmental factors (Makara-Studzińska et al., 2012). Individuals must be able to cope with stress adequately to maintain their resilient state, as this will protect them from symptoms associated with post-traumatic stress disorder, major depression, and anxiety disorders (Cathomas et al., 2019).

## Depression and resilience to stress: Inflammation and immunology system

Depression is a multifaceted cause and psychiatric factors are not the only contributing factors. Increasing evidence involving immunology aspects indicate that cytokines are mainly responsible for increasing depression or the disease evolving process (Raison et al., 2006). These molecules play a major role in inflammation and are responsible for a number of factors that increase depression risk, including a risk associated with any source of inflammation (e.g., stress, autoimmune diseases, and infections); proinflammatory cytokines are elevated in patients with depression, and can trigger depression symptoms (Wu et al., 2021).

Other specific targets may play key roles in the onset and development of depression by inducing stress. Mitochondrial damage associated with redox imbalance (ROS/RNS) promotes mitochondrial DNA (mtDNA) release and blockage of autophagic flow (accumulation) that increases release of proinflammatory cytokines and apoptotic cell death. This can cause mood disorders and neurodegenerative diseases (Freeman and Ting, 2016; Forrester et al., 2018; Andreazza et al., 2019; Moya et al., 2021).

Serotonin, noradrenaline, and glutamate are the neurotransmitters that modulate mood. Disturbances in secretion can cause mood disorders, particularly in the presence of inflammation. As a result of proinflammatory cytokines, such as TNF-α, interleukin IL-1β, and IL-6, neurotransmitter actions are dismantled, resulting in a decrease in action and an improvement in depression symptoms (André et al., 2008; Kuo et al., 2013). Research has shown that decreased interleukin-6 levels have a pro-resilient effect in stressed mice, since it is negatively correlated with social interaction behavior and repeated social defeat stress (Hodes et al., 2014).

Proinflammatory cytokines, indirectly, increase neurotoxic molecules by targeting indolamine-2,3-deoxigenaze (IDO) from tryptophan/kynurine metabolism pathways, which is majorly responsible for inflammation-induced (*in vivo* by lipopolysaccharide—LPS) deficit in memory and depression progress (Heisler and O’Connor, 2015). As a serotonin primary amino acid precursor, if tryptophan levels decrease, it influences serotonin concentration and mood maintenance.

Most dietary tryptophan is converted *via* the kynurenine pathway (KP). Under normal circumstances, tryptophan-2,3-deoxigenaze (TDO) hepatic enzyme is responsible for the initial step of the kynurenine pathway: tryptophan is converted to N-formylkynurenine and later to kynurenine. However, in a proinflammatory context (e.g., stress and depression), the extrahepatic enzyme IDO is upregulated in the periphery as well as in the brain and converts tryptophan into kynurenine (André et al., 2008). The kynurenine from this pathway produces the precursor for quinolinic acid (QUIN), an NMDA receptor agonist, which leads to increased glutamate release and, consequently, an oxidative stress state (free radical generator), a toxic environment for the CNS.

Another CNS important molecule associated with resilience and depression mood, the brain-derived neurotrophic factor (BDNF) promotes the survival of neurons by playing a role in the growth, maturation and maintenance of these cells. In the brain, the BDNF protein is found in synapses regulating synaptic plasticity, which is important for learning and memory. Krishnan et al. (2007) showed that susceptible mice had high BDNF release levels from ventral tegmental area (VTA) nucleus accumbens (NAc) area. The mesolimbic dopamine (DA) pathway is a reward circuit, in which resilient mice display control-level firing of DA neurons from VTA to NAc (Friedman et al., 2014; Han and Nestler, 2017). It seems excessive and prolonged VTA activation and dopamine release could change harmless social to aversive status. Postmortem NAc samples from human depressed patients also showed increased BDNF levels, demonstrating relationships between pre-clinical and clinical results (Krishnan et al., 2007). Furthermore, BDNF genotypes can be associated with better responses to antidepressant treatments. BDNF Val^66^Met polymorphism genotypes are associated with resilience, and cognitive psychotherapy improves resilience scores and depressive symptoms independently in MDD patients (Peters et al., 2020).

The IL-1β inhibits glutamate reuptake in the brain and enhances glutamate release, decreasing glutamate levels and BDNF concentration, thereby affecting serotonin/noradrenaline metabolism (Merali et al., 1997; Day et al., 1999; Tilleux and Hermans, 2007). Scotton et al. (2020) demonstrated that chronic unpredictable mild stress increases BDNF concentrations and prevents oxidative stress in lipids and proteins in hippocampal cells. In addition, a recent study has reported that chronic unpredictable mild stress modulates the NLRP3 complex, which causes the proteolytic cleavage of pro-IL-1β into active and secreted IL-1β, causing proinflammatory responses and, consequently, cellular damage (Wang et al., 2021).

Tryptophan metabolism and KP occur in astrocytes and microglia, which are important nervous cells involved in the inflammation process (Heisler and O’Connor, 2015). Various inflammatory sources can disrupt the blood-brain barrier (BBB) sculpted by astrocytes, leading to an increase in cytokine molecules and immune cells from peripheral regions, thereby amplifying the neuroinflammatory condition (Yang et al., 2020). The astrocyte-microglia crosstalk is a bond between these two cells that coordinates the neuroinflammatory state (Vainchtein and Molofsky, 2020), which focuses on immune responses regarding emotion and affection, resulting in altered astrocyte-microglia crosstalk (Yang et al., 2020). Furthermore, under pathological circumstances, BBB permeability increases due to the upregulation of TNFα/NFκB signaling pathways, decreasing the expression of tight junction proteins (claudins) (Günzel and Yu, 2013). The same pathway was found to be upregulated in stress-induced mice and repressing the tight junction protein claudin-5 (cldn5), but not in the unstressed control, indicating that resilient mice have a TNFα/NFκB inflammation pathway downregulated (Dudek et al., 2020). In addition to the BBB, mitochondrial disorders of microglial cells can also produce O_2_^–^, H_2_O_2_, and cytokines, triggering a central inflammatory process (Halliwell, 2006; Song and Wang, 2011; Friedman et al., 2014). The increased expression of IL-6 in mice challenged with LPS makes them more susceptible (Hodes et al., 2014).

In addition to cytokines, the natural killer cells (innate immunity) play an important role with marked decreased activity in patients with depression (Zorrilla et al., 2001). Glucocorticoid resistance in depressed individuals is associated with immune activation (Miller et al., 2002; Raison and Miller, 2011).

Although depression is characterized by high IL-6 levels, the relationship between natural killer cells and IL-6 has not been proven in humans (Pike and Irwin, 2006). However, natural killer cell-derived exosomes containing miRNA (miR-207) demonstrated antidepressant action and decreased inflammation pathways by targeting astrocytes (Li et al., 2020).

Recently, some authors concluded that repeated restraint stress modulates neuroinflammation by microglial activation and increases interleukin (IL-6) and TNF-α levels in the amygdala (Liu et al., 2018) and hippocampus (Jangra et al., 2020).

With regard to adaptive immunity, many studies have focused on cellular immunity, namely T lymphocytes, in individuals with depression. Initially, T cells counts may serve as a biomarker of suicidal behavior in youth with depression or anxiety (Amitai et al., 2022).

Specific T helper cell types are related to depression and stress states, namely, Th1, Th17 and Treg. Th1 and Th17 cells are related to depression, especially in patients with increased IL-17 levels (Davami et al., 2016). In addition, mice under repeated social distress showed a marked IL-17 increase in Th17 cells (Elkhatib et al., 2022). In other mood disorders, such as bipolar disorder, patients present with low levels of Treg cells along with immune and inflammatory imbalance (do Prado et al., 2013).

## Depression and resilience to stress: Oxidative stress

In natural processes, the body produces free radicals and activate cell signaling which are vital for immune system activation (Maes et al., 2011; Song and Wang, 2011; Muri and Kopf, 2021) and it is necessary for the protection of our body from infections caused by viruses, bacteria and fungi (Babior, 2000).

However, its excessive production may cause several forms of cellular damage to proteins, carbohydrates, lipids, and DNA, and its toxicity is involved in the development of numerous diseases. The imbalance between the production of ROS and RNS and the organism’s ability to inhibit or repair the damage caused by them through its endogenous antioxidant defenses or the use of exogenous antioxidants results in oxidative stress (Halliwell, 2011; Yao and Keshavan, 2011).

Oxidative stress is commonly associated with the pathogenesis of diseases, such as cancer, cardiovascular disease, and diabetes. Additionally, it plays a key role in brain aging, increasing the risk factors for most neurodegenerative diseases, the pathophysiology of dementia, and many psychiatric disorders, such as depression, schizophrenia, bipolar mood, and anxiety disorders (Nicolai et al., 2015; Chen and Liu, 2017; Liguori et al., 2018; Singh et al., 2019).

Lehmann et al. (2019) suggested that stress increases the ROS in microglial cells and hinders normalization after a stress situation and it is important to mention that many compounds attenuate biochemical changes caused by stress animal models through oxidative stress (Umukoro et al., 2018; Gao et al., 2019). Furthermore, repeated restraint stress augments oxidative and nitrosative damage, decreasing Na, K,-ATPase activity in the frontal cortex, which is an important region of the brain for mood control (Novaes et al., 2018).

The brain is particularly susceptible to oxidative damage because it is an organ with extremely high metabolism, with high consumption of oxygen necessary to meet the intense demand for adenosine triphosphate (ATP), in addition to containing excitatory amino acids and neurotransmitters whose metabolism produces ROS and others oxidant molecules (Maes et al., 2011; Scaini et al., 2016; Salim, 2017; Michels et al., 2018).

Neuronal membranes are particularly vulnerable to oxidation because of the large amount of unsaturated fatty acids associated with their elongated morphology. The presence of a large amount of glutamate promotes the mobilization of intracellular calcium, which interferes with ion transportation and consequently increases oxidative stress (Bouchelouche et al., 1989; Ikeda et al., 2005). Evidence indicates that astrocytes may be a target of future therapies for the modulation of oxidative stress in CNS disorders. They play a dual role in the regulation of ROS/RNS, being able to protect the CNS by removing excitatory amino acids and activating the production of the endogenous antioxidant nuclear factor erythroid 2-related factor (Nrf2) system, and also acting as a source of ROS/RNS due to the mitochondrial dysfunction (Chen et al., 2020).

The mitochondria present in CNS cells may activate signaling pathways orchestrated by mitochondrial ROS and may lead to neuroprotection or neurodegeneration. However, damage to biomolecules induced by oxidative stress has a strong potential to negatively affect normal CNS functions (Salim, 2017). Decreases in energy production due to mitochondrial damage (Scaini et al., 2016) may block autophagic flow, mitophagy (Kurz et al., 2008; Shu et al., 2019; Tripathi et al., 2021) and apoptosis (Kim et al., 2010).

Extrinsic and intrinsic factors may cause mitochondrial damage and apoptotic death of astrocytes in the cortex and hippocampus, which can lead to depression (Kong et al., 2014; Michels et al., 2018; Shu et al., 2019; Shandilya et al., 2022). However, the duality between disorder and resilience is generally associated with repair and epigenetic mechanisms (Michels et al., 2018) as both genetic and environmental factors are important for the onset of depression.

Exposure to environmental threats induces adaptive changes in neuroplasticity in the brain (Uchida et al., 2018). In animal models, an imbalance between the generation and suppression of ROS, neuroinflammation, and apoptosis is associated with the onset of depression (Kubera et al., 2011; Uchida et al., 2018) and DNA methyltransferase (DNMT) activity plays a role in depression in animals (LaPlant et al., 2010; Uchida et al., 2011). Histone modifications and DNA methylation, such as in the glial cell-derived neurotrophic factor (Gdnf) promoter, play crucial roles in the control of behavioral responses to chronic stress (Uchida et al., 2011).

Furthermore, protection mechanisms through mitochondrial gene expression, signaling, gene repair pathways (Yang et al., 2011) and epigenetics are resistance/resilience targets for depression (Michels et al., 2018; Uchida et al., 2018). We currently know that exposure to stressors can alter epigenetic markers in animal models and humans. These changes in specific genes in chromatin may be useful for diagnosing and treating mental illnesses. The question remains as to how they can be evaluated in living humans. Therefore, blood and peripheral tissue biomarkers are used (Zannas and West, 2014).

Enhancing mitochondrial function could represent an important avenue for novel therapeutics as well as genetic and epigenetic development (Manji et al., 2012). The damage and resilience caused by nutrition (Du et al., 2016), microbiota (Shandilya et al., 2022) and pharmacological treatments (Shu et al., 2019) influence mitochondrial structure and mechanisms, which may be a strategy to prevent or treat depression (Michels et al., 2018).

## Conclusion

The purpose of this review is to summarize the current knowledge regarding the neurobiology of depression and resilience. A combination of findings from clinical studies and preclinical models of mood disorders supports the role of genetics, synaptic plasticity, immune and oxidative stress in stress response. Many people around the world suffer from depression, a mood disorder that is a biologically heterogeneous disease involving several systems; Despite this, depression has a poorly understood pathophysiology. Understanding the mechanisms underlying depression could help develop new therapeutic approaches for treating or improving the quality of life of depressed patients. Future clinical studies could recruit highly resilient individuals for comparison with depressed patients and healthy controls. Increasing insight into the biology of resilience may help us to elucidate novel targets.

## Author contributions

All authors listed have made a substantial, direct, and intellectual contribution to the work, and approved it for publication.
